# Supplementary material for: WordCommentsAnalyzer: A windows software tool for qualitative research
Source: F1000Res. 2018 Sep 4;7:536. Originally published 2018 May 3. [Version 2] doi: 10.12688/f1000research.14819.2 (PMC6137411; doi:10.12688/f1000research.14819.2)
Supplement: Supplementary file 3 [file f1000research-7-17614-s0002.tgz › b2a79496-4ada-47c4-9682-a99ce867836d.zip!/Eight Iranian Health Journals/Archives of Rehabilitation_21.docx]

© Archives of Rehabilitation. This work is licensed under CC BY-NC 4.0 (https://creativecommons.org/licenses/by-nc/4.0/).

Volume & Issue: دوره 14 - شماره 3

Title: مقایسه وضوح گفتار کودکان کاشت حلزون شده، دارای سمعک و کودکان با شنوایی هنجار

http://rehabilitationj.uswr.ac.ir/article-1-1286-fa.html

هدف: هدف این پژوهش مقایسه وضوح گفتار کودکان کاشت حلزون شده، دارای سمعک و کودکان با شنوایی هنجار در شهرستان‌های تهران بود. روش بررسی: در این پژوهش تحلیلی و مقایسه‌ای، شصت کودک پنج تا هفت سال شرکت کردند. آن‌ها به سه گروه بیست‌نفری تقسیم شدند؛ به‌نحوی‌که گروه اول و دوم به‌ترتیب، از کودکان کاشت حلزون شده و دارای سمعک بودند که به روش نمونه‌گیری در دسترس انتخاب شدند؛ درحالی‌که گروه سوم، شنوایی هنجار داشتند و به روش تصادفی انتخاب شدند. ابزارهای استفاده‌شده در این پژوهش، آزمون هوشی ریون و مقیاس تعیین سطح وضوح گفتار بود. اطلاعات جمع‌آوری‌شده با استفاده از تحلیل پراکنش یک‌طرفه و آزمون تعقیبی شفه تحلیل شد. یافته‌ها: نتایج نشان داد که میانگین نمرات وضوح گفتار کودکان با شنوایی هنجار به طور معناداری بیشتر از دو گروه دیگر بود (۰/۰۵>P)، ولی میانگین نمرات وضوح گفتار کودکان کاشت حلزون شده و کودکان دارای سمعک تفاوت معناداری نداشت (۰/۰۵>P). نتیجه‌گیری: وضوح گفتار در کودکان با شنوایی هنجار بهتر از کودکان کاشت حلزون شده و دارای سمعک بود ولی از آن جایی که وضوح گفتار یک پدیده پیچیده و چند بُعدی است، به برنامه توانبخشی ویژه‌ای نیاز است تا تحول بیشتری در مهارت گفتاری کودکان کاشت حلزون شده یا دارای سمعک ایجاد شود.

Title: بررسی تأثیر تمرینات ثبات مرکزی بر وضعیت تعادل دانش‌آموزان پسر کم‌توان ذهنی آموزش‌پذیر

http://rehabilitationj.uswr.ac.ir/article-1-1208-fa.html

هدف: هدف از تحقیق حاضر بررسی تأثیر تمرینات ثبات مرکزی بر وضعیت تعادل دانش‌آموزان پسر کم‌توان ذهنی آموزش‌پذیر بود.  روش بررسی: پژوهش از نوع کاربردی بود و در آن، از طرح شبه‌آزمایشیِ پیش‌آزمون و پس‌آزمون با گروه کنترل استفاده شد. نمونۀ آماری ۳۱ دانش‌آموز پسر عقب‌ماندۀ ذهنی بودند که در دو گروه قرار گرفتند: گروه اول (کنترل): ۱۴ نفر با میانگین سن ۳/۰۲ ± ۱۱/۰۷ سال و قد ۷/۸۶±۱۵۲ سانتی‌متر و وزن ۸/۰۸±۴۴/۰۷ کیلوگرم؛ گروه دوم: ۱۷ نفر تجربی با میانگین سن ۱/۹۵±۱۱/۲۳ سال و قد ۷/۰۷±۱۴۷ سانتی‌متر و وزن ۴/۸۵±۳۸/۱۱ کیلوگرم. آزمودنی‌ها ازطریق نمونه‌گیری در دسترس (تصادفی) انتخاب شدند و اطلاعات فردی آن‌ها مانند قد، وزن، هوش و سوابق پزشکی، با استفاده از پروندۀ پزشکی‌شان به‌دست آمد. برنامۀ تمرینی گروه تجربی شامل تمرین‌های مرتبط با ثبات مرکزی بود. آزمودنی‌ها تمرینات را در شش هفته (سه بار در هفته) انجام دادند. در سه هفتۀ اول، در دو سِت با پنج تکرار و در سه هفتۀ دوم، در دو سِت با ده تکرار انجام دادند و برای ارزیابی تعادل پویا از تست تعدیل‌شدۀ ستارۀ Y استفاده شد. برای تحلیل داده‌ها از آزمون‌های آماری تی مستقل و هم‌بسته استفاده شد.  یافته‌ها: براساس نتایج، اختلاف معنی‌داری در جهات خلفی خارجی و خلفی داخلی در گروه تجربی پس از تمرینات وجود دارد (۰/۰۵&leP).  نتیجه‌گیری: افراد مبتلا به کم‌توانی ذهنی درمقایسه‌ با افراد طبیعی در رشد حرکتی تأخیر دارند که به‌نظر می‌رسد باعث کاهش تعادل در آن‌ها می‌شود. این پژوهش باتوجه‌به ضعف تعادل در کم‌توانانان ذهنی و اهمیت تعادل در فعالیت‌های روزانه و تأثیر تمرینات ثبات مرکزی بر تعادل نشان داد که این تمرینات باعث بهبودی تعادل در این افراد می‌شود. مربیان و معلمان نیز می‌توانند از تمرینات ثبات مرکزی برای افراد کم‌توان ذهنی استفاده کنند.

Title: بررسی علل ژنتیکی ناتوانی ذهنی (آتوزومی و وابسته به ایکس) در شهرستان اهواز

http://rehabilitationj.uswr.ac.ir/article-1-1213-fa.html

هدف: ناتوانی ذهنی در ۷٠ درصد موارد علل ژنتیکی دارد. این مطالعه به‌منظور شناسایی علل ژنتیکی ناتوانی ذهنی و همچنین، کمک به مشاورۀ ژنتیک و تشخیص پیش از تولد این بیماری، در شهرستان اهواز انجام شد. روش بررسی: در این پژوهش توصیفی که از نوع مطالعات مقطعی‌کاربردی است، با کمک سازمان بهزیستی شهرستان اهواز، پس از بررسی ١۸۳ پرونده از خانوارهای تحت‌ پوشش این سازمان، ۴٠ خانوادۀ واجد شرایط انتخاب شدند که دارای دو فرزند مبتلا به ناتوانی ذهنی ارثی یا بیشتر بودند (۶۲ پسر و ۴۲ دختر) و پزشک بیماری آن‌ها را تأیید کرده بود. سپس، برای بررسی‌های مولکولی در خانواده‌های انتخاب‌شده، از خون تمام افراد سالم و مبتلا نمونه‌گیری شد. همچنین، برای همۀ بیمارانی که در معاینات بالینی آن‌ها اختلالات دیسمورفیک مشاهده گردید،کاریوتایپینگ انجام شد. یافته‌ها: در بررسی کروموزومی به روش کاریوتایپ، در هیچ‌یک از نمونه‌هاناهنجاری‌های کروموزومی مشاهده نشد. در بررسی خانواده‌ها ازلحاظ ابتلا به نشانگان ایکس شکننده به‌وسیلۀ آزمون‌های تشخیصی پی‌سی‌آر و ساترن‌بلات، ۳ خانوار (۷/۵ درصد) مبتلا به نشانگان ایکس شکننده بودند. همچنین، ۳ خانواده (۷/۵ درصد) دارای فرزندان میکروسفال از نوع اتوزومی مغلوب اولیه بودند که پس از بررسی‌های آنالیز پیوستگی در آن‌ها، هیچ‌یک از خانواده‌ها به جایگاه‌های مربوط (ام.سی.پی.اچ) پیوستگی نشان نداد. علاوه‌براین، در بررسی خانوار‌ها ازلحاظ ابتلا به اختلالات متابولیک به روش اسپکترومتری، هیچ‌گونه اختلال متابولیک مشاهده نشد. ۲ خانواده که دچار عقب‌ماندگی ذهنی از نوع غیرسندرمی و غیرمیکروسفال بودند، طرح توارث اتوزوم غالب را نشان دادند. نتیجه‌گیری: براساس نتایج به‌دست‌آمده در این بررسی، ناتوانی ذهنی بسیار هتروژن است و عقب‌ماندگی ذهنی با توارث اتوزومی مغلوب به‌همراه کاهش در اندازۀ دور سر (میکروسفالی) با ۷/۵ درصد و نشانگان ایکس شکننده با ۷/۵ درصد، از علل شایع عقب‌ماندگی ذهنی ژنتیکی در این مطالعه در شهرستان اهواز است.

Title: اثربخشی آموزش رفتارهای اجتماعی بر بهبود مهارت‌های اجتماعی نوجوانان کم‌توان ذهنی

http://rehabilitationj.uswr.ac.ir/article-1-1263-fa.html

هدف: هدف این پژوهش، بررسی میزان اثربخشی آموزش رفتارهای اجتماعی بر بهبود مهارت‌های اجتماعی نوجوانان دختر کم‌توان ذهنی ۱۴‌تا‌۱۸ساله در استان مازندران است. روش بررسی: در مطالعۀ شبه‌آزمایشی حاضر با طرح پیش‌آزمون-پس‌آزمون، با گروه کنترل و با استفاده از روش نمونه‌گیری تصادفی‌خوشه‌ای و چندمرحله‌ای، چهل نوجوان دختر کم‌توان ذهنی ۱۴تا۱۸ساله، انتخاب و به‌طور تصادفی در گروه آزمایش و گروه کنترل جایگزین شدند، به‌طوری‌که هر گروه بیست نفر بود. به اعضای گروه‌ آزمایش، رفتارهای اجتماعی در هشت جلسۀ نود‌دقیقه‌ای در یک‌ماه، آموزش داده شد؛ ولی به گروه کنترل آموزشی داده نشد. ابزار جمع‌آوری اطلاعات، آزمون هوشی ریون و مقیاس مهارت‌های اجتماعی ماتسون بود. داده‌های به‌دست‌آمده با استفاده از تحلیل کوواریانس چند‌متغیری تحلیل شدند.  یافته‌ها: نتایج پژوهش حاضر نشان داد که میانگین نمره‌های مهارت‌های اجتماعی گروه آزمایش به‌طور معناداری بیشتر از گروه کنترل بود (۰/۰۵>P). همچنین، میانگین نمره‌ها در همۀ خرده‌مقیاس‌های مهارت‌های اجتماعی، شامل مهارت‌های اجتماعی مناسب، رفتارهای غیراجتماعی، پرخاشگری و تکانشی، برتری‌طلبی و اطمینان زیاد به‌خود، رابطه با همسالان، در مرحلۀ پس‌‌آزمون، در گروه آزمایش به‌طور معناداری افزایش یافته بود (۰/۰۵>P).  نتیجه‌گیری: برنامۀ آموزشی رفتار اجتماعی، باعث بهبود مهارت اجتماعی در نوجوانان کم‌توان ذهنی شد؛ بنابراین برنامه‌ریزی برای آموزش رفتار اجتماعی به این نوجوانان اهمیت ویژه‌ای دارد.

Title: اثر طول گفته بر روانی گفتار خودانگیخته کودکان و بزرگسالان لکنتی فارسی زبان

http://rehabilitationj.uswr.ac.ir/article-1-770-fa.html

هدف: چگونگی ارتباط میان طول گفته با ناروانی های لکنت در سال های اخیر مورد توجه محققین قرار گرفته است. هدف این پژوهش نیز، بررسی تاثیر طول گفته بر میزان ناروانی گفتار کودکان و بزرگسالان لکنتی فارسی زبان در تکلیف گفتار خودانگیخته می باشد. نتایج بدست آمده می تواند جهت شناخت بیشتر ماهیت لکنت کودک و بزرگسال و نیز یافتن شیوه های مناسب تر برای درمان آن کاربرد داشته باشد. روش بررسی: این مطالعه توصیفی- تحلیلی از نوع مقطعی است در این مطالعه از روش نمونه گیری آسان استفاده شده است به این ترتیب که 15 بزرگسال لکنتی تک زبانه و فارسی زبان 15 سال به بالا و 15 کودک لکنتی تک زبانه و فارسی زبان 4 تا 6 ساله می باشند. پس از تائید لکنت به کمک آزمون ابزار SSI-3، نمونه ای 30 دقیقه ای از گفتار کودکان و بزرگسال ضبط و فیلمبرداری شد. سپس ناروانی و طول گفته های هر فرد مورد بررسی قرار گرفت. اطلاعات بدست آمده توسط نرم افزار spss و با استفاده از آزمون آماری تی زوج مورد تجزیه و تحلیل قرار گرفت. یافته‌ها: میانگین طول جملات ناروان نسبت به روان به طور معناداری در هر دو گروه بیشتر بود. (P=0/0) نتیجه‌گیری: در تکلیف گفتار خودانگیخته، با افزایش طول گفته، میزان ناروانی گفتار کودکان و بزرگسالان لکنتی افزایش معناداری می‌یابد.

Title: بررسی تأثیر پاشنه‌های استاندارد و شیب دار در کفش طبی بر مولفه عمودی نیروی عکس‌العمل زمین در هنگام راه رفتن در افراد سالم

http://rehabilitationj.uswr.ac.ir/article-1-1285-fa.html

هدف: در راه رفتن طبیعی، نیروهای عمودی عکس العمل زمین بصورت تکراری به سیستم اسکلتی– عضلانی بدن منتقل می-شود. بزرگی این نیرو‌ها در زمان برخورد پا با زمین، یک عامل مهم در ایجاد ضایعات ناشی از "استفاده بیش از حد"می‌باشد. هدف این مطالعه بررسی تاثیر دو نوع پاشنه استاندارد و شیب دار در کفش طبی بر مولفه‌های عمودی نیروی عکس‌العمل زمین درهنگام راه رفتن در افراد سالم بود. روش بررسی: در این مطالعه، 30 فرد بزرگسال بصورت غیراحتمالی ساده شرکت داشتند. افراد در سه حالت پابرهنه، استفاده از کفش طبی با پاشنه استاندارد و کفش طبی با پاشنه شیب دار در یک مسیر 8 متری راه رفتند. داده‌های نیروی عمودی عکس العمل زمین در مرحله ابتدایی فاز ایستایی راه رفتن با استفاده از صفحه نیروسنج کیستلر جمع‌آوری شد. یافته‌ها: نتایج این تحقیق نشان داد که نیروی ضربه‌ای در حالت پابرهنه در مقایسه با کفش طبی با پاشنه‌های استاندارد و شیب دار افزایش معنادار یافته است (P˂0/0001). قله اول نیروی عمودی عکس العمل زمین و نرخ بارگذاری در کفش طبی با پاشنه شیب دار در مقایسه با کفش طبی با پاشنه استاندارد کاهش معناداری یافته است (P˂0/05). نتیجه‌گیری: استفاده از پاشنه شیب دار با تغییر فاصله محل اثر نیروی عکس العمل زمین نسبت به محور مفصل مچ، باعث تسهیل راکر اول، کاهش قله اول نیروی عمودی عکس العمل زمین و نرخ بارگذاری گردید. با توجه به اثرات آسیب-رسان بارهای وارده به اندام تحتانی بویژه در مرحله ابتدایی راه رفتن، می‌توان هنگام تجویز کفش طبی در بیماران ارتوپدی از این نوع پاشنه نیز استفاده کرد.

Title: تأثیر توانبخشی قلبی مبتنی بر بیمارستان بر کیفیت زندگی بیماران مبتلا به سکته قلبی بیمارستان بقیه ا... تهران در سال 1389

http://rehabilitationj.uswr.ac.ir/article-1-1259-fa.html

هدف: هدف این تحقیق بررسی تأثیر برنامه توانبخشی قلبی مبتنی بر بیمارستان بر روی کیفیت زندگی بیماران مبتلا به سکته قلبی بیمارستان بقیه ا... در سال 1389 بوده است. روش بررسی: در این پژوهش نیمه تجربی 50 بیمار مبتلا به سکته قلبی بر اساس شرایط ورود به مطالعه انتخاب شدند (25 نفر در گروه مداخله و 25 نفر در گروه کنترل). روش نمونه گیری در این پژوهش برای گروه مداخله به صورت پشت سر هم و برای گروه کنترل به صورت تصادفی جور شده با اعضای گروه آزمایش (بر اساس سن، جنس و سطح تحصیلات) انجام گرفت. اطلاعات با استفاده از پرسشنامه دموگرافیک، کیفیت زندگی مک نیو و مصاحبه جمع آوری گردید. گروه مداخله طی 20 جلسه تحت آموزش نظری و عملی برنامه توانبخشی قلبی در بیمارستان مشتمل بر آشنایی با سکته قلبی، عوارض و درمان آن، عوامل خطرزای بیماری و کنترل آنها قرار گرفت. برای بیماران گروه کنترل نیز مداخله ای صورت نگرفت. داده های پژوهش با استفاده از نرم افزار spss مورد تجزیه و تحلیل قرار گرفت. جهت بررسی تفاوت نمرات گروه مداخله و کنترل آزمون تی زوجی و تی مستقل استفاده شده است. یافته‌ها: یافته های پژوهش نشان دهنده افزایش کیفیت زندگی بیماران گروه مداخله پس از انجام توانبخشی قلبی در بیمارستان می باشد. می توان تفاوت میانگین قبل و بعد کیفیت زندگی در ابعاد جسمی (P<0/05)، روحی روانی (P<0/05)، اجتماعی (P<0/05) و کل (P<0/05) در گروه مداخله را به اجرای برنامه توانبخشی قلبی مبتنی بر بیمارستان نسبت داد. نتیجه‌گیری: نتیجه گیری می شود که برنامه ‌توانبخشی قلبی مبتنی بر بیمارستان بر کیفیت زندگی بیماران مبتلا به سکته قلبی مؤثر است.

Title: بررسی روایی و پایایی آزمون یادگیری شنوایی–کلامی ری در کودکان دچار اختلال بیش فعالی نقص توجهی

http://rehabilitationj.uswr.ac.ir/article-1-1278-fa.html

هدف: افزایش ضرورت ارزیابی حافظه در کار بالینی در ایران و همچنین عدم دسترسی به یک تکلیف جامع و استانداردسازی شده بر اساس فرهنگ و زبان فارسی در جمعیت کودکان، کاربرد نسخه فارسی مقیاس عصب روان شناختی رایج را برای ارزیابی حافظه و یادگیری کلامی، یعنی آزمون حافظه شنوایی کلامی ری، در این گروه ایجاب می کند. به همین علت، هدف پژوهش حاضر بررسی روایی و پایایی آزمون حافظه شنوایی-کلامی ری در کودکان فارسی زبان مبتلا به اختلال بیش فعالی نقص توجه بود. روش بررسی: مطالعه حاضر روی 46 کودک هنجار و 37 کودک مبتلا به اختلال بیش فعالی نقص توجه راست دست در محدوده سنی 7 تا 11 سال از دو جنس با شنوایی هنجار، با تکمیل نسخه فارسی آزمون حافظه شنوایی- کلامی ری و آزمون ظرفیت توجه شنوایی پیوسته انجام شد. یافته‌ها: در تعیین روایی همگرا، بین نتایج آزمون ری در سه مرحله میانگین، یادآوری بلافاصله، یادآوری تاخیری و شاخص امتیاز کل (تعداد کل خطاها) آزمون ظرفیت توجه شنوایی پیوسته همبستگی منفی معناداری دیده شد (r=-0/536, P=0/001). بررسی روایی تفکیکی با مقایسه امتیازات آزمون بین دو گروه کودکان هنجار و کودکان دچار اختلال نقص توجه بیش فعالی، تفاوت معنادار یافته های آن ها را در کلیه مراحل آزمون نشان داد (0/001>P). همچنین بررسی پایایی آزمون با اجرای نسخه دوم آزمون روی 23 کودک هنجار، همبستگی بالایی بین نتایج دو نسخه آزمون نشان داد. نتیجه‌گیری: نسخه فارسی آزمون حافظه شنوایی- کلامی ری از روایی و پایایی خوبی برخوردار است و می توان از آن برای بررسی نقص حافظه شنوایی-کلامی در کودکان مبتلا به اختلال بیش فعالی نقص توجهی استفاده کرد.

Title: ارزیابی وضعیت عملکرد اجرایی بیماران مبتلا به اسکیزوفرنیا و مقایسه آنان با مبتلایان به اختلال دو قطبی I

http://rehabilitationj.uswr.ac.ir/article-1-668-fa.html

هدف: پژوهشهای گوناگونی برای ارزیابی وضعیت عملکرد شناختی بیماران مبتلا به اسکیزوفرنیا و اختلال دو قطبی I و مقایسه آنها با یکدیگر انجام گرفته است. ولی انجام چنین پژوهشی در بیماران سرپائی در ایران سابقه چندانی ندارد. روش بررسی: دو گروه پنجاه نفری از بیماران سرپائی مراجعه کننده به درمانگاه های بیمارستان روزبه که براساس ملاکهای DSM-IV-TR مبتلا به اسکیزوفرنیا و اختلال دو قطبی I (که دست کم به مدت سه ماه در وضعیت یوتایمیک) بوده‌اند، به صورت نمونه های در دسترس انتخاب شده و مورد بررسی قرار گرفتند. دو گروه آزمودنیها از نظر سه متغیر اصلی که براساس منابع ، بیشترین سهم را در وضعیت شناختی بیماران ممکن است داشته باشند یعنی سن ، میزان تحصیلات، طول مدت ابتلاء به بیماری به دقت همسان سازی شدند. برای هر یک از آزمودنیها ، آزمون دسته بندی کارتهای ویسکانسین انجام گرفته و نتایج با استفاده از روشهای آمار توصیفی و آمار تحلیلی با یکدیگر مقایسه شد. یافته‌ها: میانگین نتایج آزمون دسته‌بندی کارتهای ویسکانسین در هر دو گروه مبتلایان به اسکیزوفرنیا و اختلال دو قطبی به طور معنی‌داری پایین‌تر از جمعیت نرمال بود (P<0/05) و سطح عملکرد شناختی در بیماران اسکیزوفرنیا به طور معنی‌داری نسبت به بیماران دو قطبی پایین‌تر بود(P<0/05). نتیجه‌گیری: عملکرد اجرایی در مبتلایان به اسکزوفرنیا ضعیفتر از مبتلایان به اختلال دو قطبی I است.

Title: مداخله ارگونومی در واحد کوره بلند یک شرکت فولاد

http://rehabilitationj.uswr.ac.ir/article-1-1207-fa.html

هدف: ناراحتی‌های اسکلتی–عضلانی بخش عمده‌ای از بیماری‌های شغلی را در محیط‌های کاری به خود اختصاص می‌دهند، پیشگیری از بروز این ناراحتی‌ها مستلزم ارزیابی و اصلاح وضعیت‌های کاری با استفاده از روش‌های تحلیل شغلی ارگونومی می‌باشد. هدف این مطالعه، بررسی علت شیوع اختلالات اسکلتی– عضلانی در کارگران واحد کوره بلند و اجرای اقدام اصلاحی جهت کاهش میزان شیوع این اختلالات می‌باشد. روش بررسی: در این مطالعه ارزیابی بر روی 24 نفر کارگر واحد کوره بلند به روش سرشماری انجام شد، مستندات پزشکی این افراد بررسی و پرسشنامه نوردیک توسط آنان تکمیل گردید. وظیفه مته‌زنی و اکسیژن‌‎کاری توسط روش ارزیابی سریع کل بدن (REBA) ارزیابی گردید و به کارگران آموزش‎های لازم داده شد. در وظیفه اکسیژن‌‎کاری برای کل 24 نفر طراحی مجدد ایستگاه کار صورت گرفت و عمل مته‎زنی مکانیزه شد. و وظیفه کارگران دارای اختلالات اسکلتی عضلانی تغییر داده شد. ارزیابی مجدد توسط روش REBA و پرسشنامه نوردیک صورت گرفت. یافته‌ها: بر طبق نتایج پرونده‌های پزشکی و پرسشنامه نوردیک حدود 5/37 درصد کارگران اختلالات اسکلتی–عضلانی داشتند؛ امتیاز نهایی REBA برای وظیفه مته‎زنی 11 و برای اکسیژن‌کاری 10 بدست آمد. بعد از طراحی مجدد ایستگاه کار، امتیاز نهایی REBA برای وظیفه اکسیژن‌کاری 5بدست آمد و با مکانیزه نمودن عمل مته‎زنی، وظیفه مته‎زنی حذف گردید. با اجرای اقدامات اصلاحی میزان اختلالات اسکلتی– عضلانی 5/17 درصد کاهش یافت. نتیجه‌گیری: با اجرای اقدامات اصلاحی در واحد کوره طبق روش REBA سطح ریسک ابتلا به اختلالات اسکلتی- عضلانی کاهش یافت و میزان شیوع اختلالات اسکلتی-عضلانی نیز به میزان قابل توجهی کاهش یافت. کلید واژه‌ها: مداخله ارگونومی، ناراحتی‌های اسکلتی – عضلانی، REBA ، پرسشنامه نوردیک، فولاد.

Title: اثرات تب و تشنج ساده بر تغییرات مولکولی آپوپتوزیس در هیپوکامپ نوزادان موش‌های صحرایی

http://rehabilitationj.uswr.ac.ir/article-1-1253-fa.html

هدف: تب و تشنج شایع‌ترین اختلال تشنجی دوران کودکی است و در 5-2 درصد از کودکان رخ می‌دهد. تب و تشنج ساده معمولاً به عنوان یک اختلال بی ضرر محسوب می‌شود، با این وجود شواهد نشان می‌دهند که تب و تشنج ساده با اختلالات هیپوکامپ در بزرگسالی مرتبط است. این مطالعه با هدف اندازه‌گیری سطح پروتئین‌های Bcl-2 و Bax در هیپوکامپ نوزادان موش‌های صحرایی بعد از القاء تب و تشنج ساده طراحی گردید. روش بررسی: در این مطالعه تجربی، القاء تب و تشنج با استفاده از حمام آب گرم در نوزادان نر 22 روزه‌ی موش‌های صحرایی شبیه سازی شد. حیوانات براساس وجود یا عدم وجود رفتارهای تشنجی به دو گروه تقسیم شدند: هیپرترمی همراه با تشنج (n=10) و هیپرترمی بدون تشنجn=10) ) جهت کنترل اثرات استرس محیطی یک گروه شم-کنترل (n=10) نیز به مطالعه افزوده شد. هیپوکامپ موش‌های صحرایی در روز 15 بعد از هیپرترمی خارج و بیان پروتئین‌های Bcl-2 و Bax با استفاده از تکنیک وسترن بلاتینگ اندازه‌گیری گردید. یافته‌ها: نتایج نشان داد که سطح پروتئین Bcl-2 در هیپوکامپ گروه هیپرترمی همراه تشنج به طور معنی‌داری کمتر از گروه کنترل-شم و هیپرترمی بدون تشنج بود؛ از طرف دیگر، سطح پروتئین Bax در گروه هیپرترمی همراه تشنج در مقایسه با گروه کنترل-شم و هیپرترمی بدون تشنج به طور قابل توجهی بیشتر بود. نتیجه‌گیری: القاء تب و تشنج ساده با تغییر در سطح پروتئین‌های Bcl-2 و Bax مرتبط است که نشانگر پیشرفت آپوپتوز سلولی در هیپوکامپ نوزادان موش‌های صحرایی است که حداقل تا 15 روز قابل اندازه‌گیری است.

Title: بررسی تغییرات سفتی عضلانی بر اساس شاخص های بیو مکانیکی در مفصل آرنج بیماران سکته مغزی

http://rehabilitationj.uswr.ac.ir/article-1-1119-fa.html

هدف: در تحقیق حاضر تلاش شد که روند تغییرات سفتی عضلانی بر اساس معیارهای بیومکانیکی در مفصل آرنج بیماران سکته مغزی، به ازاء افزایش سرعت حرکت غیر فعال مفصل آرنج و افزایش میزان معیار MAS، بررسی شود. روش بررسی: شاخصهای بیو مکانیکی ویسکوز و الاستیک در بخشهای مختلف از نمودار گشتاور زاویه حرکت غیر فعال فلکشن و اکستنشن مفصل آرنج در 15 بیمار سکته مغزی و 15 فرد سالم توسط دینامومتر ایزوکینتیک سایبکس (CYBEX) ثبت گردید و برنامهای در محیط MATLAB توسعه یافت که قادر به محاسبهی شاخصها در هر بازه بود. بیماران را بر اساس شدت معیار کیفی سفتی عضلانی (MAS) به 3 گروه 1، 2 و 3 تقسیم کرده و شاخص های مورد نظر برای گروه بیمار و گروه کنترل در 4 سرعت 15 ،45 ،75 و 120 درجه بر ثانیه محاسبه گردید. یافته‌ها: در خصوص شاخص های سفتی ویسکوالاستیک و سفتی ویسکوز، افزایش میزان شاخص های مربوطه به ازاء افزایش سرعت حرکت و میزان معیار MAS در بین کلیه نمونه ها مشاهده شد و در خصوص شاخص سفتی الاستیک، افزایش نا منظم شاخص مربوطه به ازاء افزایش سرعت حرکت در بین نمونه ها ی گروه 2 و 3 به وضوح مشاهده شد همچنین با افزایش میزان معیار MAS اثر شاخص مربوطه در رتبه های بالایی MAS کاهش یافت. نتیجه‌گیری: مشاهده شد که با افزایش سرعت حرکت و میزان معیار MAS، اثر سفتی ویسکوز در عضلات افزایش یافته، به این معنی که سفتی ایجاد شده در اندام درگیر در حرکت، در بیماری CVA بیشتر به دلیل سفتی عضلات و ناشی از سفتی ویسکوز بوده است.

Title: بررسی تأثیر ارتز دارای بخش حمایت قوس طولی داخلی بر توزیع فشار کف پا در زنان مبتلا به بدشکلی خفیف و متوسط هالوکس ولگوس

http://rehabilitationj.uswr.ac.ir/article-1-1275-fa.html

هدف: هالوکس ولگوس از متداول‌ترین اختلالات پنجه پا بوده که سبب افزایش فشار کف پایی زیر سر متاتارس اول و انگشت شست می‌شود. هدف از انجام این مطالعه بررسی تأثیر ارتز دارای حمایت قوس طولی داخلی بر توزیع فشارهای کف پایی در افراد مبتلا به بدشکلی خفیف و متوسط هالوکس ولگوس بود. روش‌ بررسی: در این مطالعه شبه تجربی 16 زن دچار هالوکس ولگوس از نمونه‌‌های در دسترس انتخاب شدند. فشار کف پایی با استفاده از دستگاه پدار در هشت منطقه آناتومیکی با و بدون ارتز دارای حمایت قوس طولی داخلی به صورت آنی و همچنین پس از یک ماه استفاده اندازه‌گیری شد. داده‌های حاصل با استفاده از آزمون آنالیز واریانس سنجش مکرر تحلیل شدند. یافته‌ها: نتایج نشان داد که ارتز دارای حمایت قوس طولی داخلی پس از یک ماه استفاده، موجب کاهش فشار در ناحیۀ شست (P<0/019)، متاتارس اول و متاتارس‌های سوم تا پنجم (P<0/001) و افزایش فشار در ناحیه داخلی میانی پا (P<0/001) نسبت به حالت کفش بدون حمایت قوس طولی داخلی شده است. نتیجه‌گیری: ارتز دارای حمایت قوس طولی داخلی موجب کاهش فشار در ناحیه جلوی پنجه و افزایش فشار ناحیه داخلی میانی پا شده است. در نتیجه سبب طبیعی‌تر کردن توزیع فشار کف پای افراد دچار هالوکس ولگوس شد. بنابراین می‌توان این ارتز را یکی از گزینه‌های درمانی موثر برای جلوگیری از پیشرفت در مراحل شروع این عارضه تلقی کرد.

Title: بررسی اثر تکلیف تکرار ناکلمه بر برخی شاخصهای زبانی در کودکان با آسیب زبانی ویژه

http://rehabilitationj.uswr.ac.ir/article-1-1177-fa.html

هدف: در سالهای اخیر مقالات متعددی به وجود ارتباط بین آسیب زبانی ویژه و ضعف معنادار در تکرار ناکلمه پرداخته اند. هدف از پژوهش حاضر بررسی اثر مداخله ای تکلیف تکرار ناکلمه بر برخی از شاخصهای زبانی مرتبط در کودکان با آسیب زبانی ویژه می باشد. روش بررسی: در این مطالعه تک موردی با خط پایه چندگانه، اثر مداخله ی تکلیف تکرار ناکلمه بر برخی شاخصهای زبانی مرتبط، در چهار کودک 5/6 تا 5/7 ساله دارای آسیب زبانی ویژه که به صورت در دسترس انتخاب شده بودند بررسی شد. شاخصهای مورد مطالعه در پژوهش حاضر شامل میانگین طول گفته و درصد کاربرد انواع تکواژهای قاموسی، دستوری و تصریفی بوده است و برای تعیین اثربخشی این تکلیف از شاخص اندازه اثر استفاده شد. یافته‌ها: اندازه اثر مداخله در افزایش میانگین طول گفته در هر چهار آزمودنی بالا بود. این برنامه درصد تکواژهای قاموسی در سه مورد افزایش یافته و در مورد یکی از آزمودنی ها مداخله بر این شاخص بی اثر بود. مداخله بر درصد تکواژهای دستوری درسه مورد سبب افزایش شاخص و در یک مورد باعث کاهش آن شده بود. شاخص درصد تکواژهای تصریفی در سه آزمودنی کاهش و یک مورد افزایش را نشان داد. نتیجه‌گیری: به نظر می رسد مداخله ی تکرار ناکلمه شاخصهای زبانی را در گروه آسیب زبانی ویژه بهبود بخشیده است. در نتیجه بنا بر یافته های این پژوهش استفاده از تکلیف تکرار ناکلمه در گروه آسیب زبانی ویژه پیشنهاد می شود.

Title: بررسی قابلیت الکترومیوگرافی سطحی در ارزیابی خستگی عضلات پارااسپینال بدنبال مداخلات درمانی در بیماران مبتلا به کمردرد مزمن: مروری سیستماتیک بر مطالعات گذشته

http://rehabilitationj.uswr.ac.ir/article-1-1332-fa.html

هدف: ارزیابی استقامت عضلات ناحیه کمر در بیماران مبتلا به کمردرد مزمن از اهمیت بالای برخوردار می باشد. مطالعات زیادی نشان داده اند که الکترومیوگرافی سطحی شایستگی ارزیابی خستگی عضلانی و تغییرات آن را با استفاده از طیف فرکانس دارا می باشد. هدف مطالعه حاضر مروری سیستماتیک بر ارزیابی تغییرات خستگی عضلات پارااسپینال بدنبال مداخلات درمانی در بیماران مبتلا به کمردرد مزمن با استفاده از الکترومیوگرافی سطحی بوده است. روش بررسی: جستجوی جهت مطالعات منتشر شده از سال ۲۰۰۰ تا ۲۰۱۲ در سایتهای علمی PubMed، ScienceDirect ،OVID ، CINAHL و MEDLINE انجام شد. کلمات "عضلات پارااسپینال"، " خستگی"، "کمردرد مزمن"، " مداخلات درمانی"، " ورزش"، " استقامت" و " الکترومیوگرافی سطحی" بعنوان کلمات کلیدی مورد استفاده قرار گرفت. یافته‌ها: نتایج جستجو 158 مطالعه را با کلمات کلیدی فوق الذکرمشخص نمود. تعداد هشت مطالعه براساس معیارهای ورود و خروج مطالعات مرتبط بوده اند. در این مطالعات تفاوت های زیادی در متدولوژی، شاخص های مورد اندازه گیری قبل و بعد از مداخلات، حجم نمونه، روش اجرا و پارامترهای الکترومیوگرافی دیده شد. نتیجه‌گیری: نتایج نشان می دهد که شواهد قوی جهت حمایت از شایستگی الکترومیوگرافی سطحی در ارزیابی خستگی عضلات پارااسپینال بدنبال مداخلات درمانی در بیماران مبتلا به کمردرد مزمن وجود ندارد.
